# Evolution of virulence: triggering host inflammation allows invading pathogens to exclude competitors

**DOI:** 10.1111/j.1461-0248.2007.01125.x

**Published:** 2008-01

**Authors:** Sam P Brown, Ludovic Le Chat, François Taddei

**Affiliations:** 1 Section of Integrative Biology, University of Texas at Austin Austin, TX 78712, USA; 2 Department of Zoology, University of Oxford South Parks Rd, Oxford OX1 3PS, UK; 3 London School of Hygiene & Tropical Medicine Keppel Street, London WC1E 7HT, UK; 4 University of Paris 5, Faculty of Medicine, INSERM U571, 156 Rue de Vaugirard, F-75730 Paris 15, France

**Keywords:** Allelopathy, gut pathogens, host manipulation, multiple infection, niche construction, parasite epidemiology, rock paper scissors, virulence, within-host dynamics

## Abstract

Virulence is generally considered to benefit parasites by enhancing resource-transfer from host to pathogen. Here, we offer an alternative framework where virulent immune-provoking behaviours and enhanced immune resistance are joint tactics of invading pathogens to eliminate resident competitors (transferring resources from resident to invading pathogen). The pathogen wins by creating a novel immunological challenge to which it is already adapted. We analyse a general ecological model of ‘proactive invasion’ where invaders not adapted to a local environment can succeed by changing it to one where they are better adapted than residents. However, the two-trait nature of the ‘proactive’ strategy (provocation of, and adaptation to environmental change) presents an evolutionary conundrum, as neither trait alone is favoured in a homogenous host population. We show that this conundrum can be resolved by allowing for host heterogeneity. We relate our model to emerging empirical findings on immunological mediation of parasite competition.

## Introduction

Food-borne microbial pathogens routinely face a tremendous ecological challenge: how to invade an occupied niche? From a microbial perspective, host surfaces are rich, competitive environments, supporting up to 10^14^ bacteria in the case of the human gut (Savage 1977). This resident diversity can aid the host in combating invasive pathogens, for instance commensal gut flora reduce host susceptibility to enteropathogenic bacteria (Hudault *et al.* 2001; Dillon *et al.* 2005). If an invader is at a disadvantage in the context of the resident’s environment, one solution is to modify the environment into a configuration favouring the invaders. Here, we ask whether invader-triggered environmental change can facilitate invasion by emptying the niche of former residents.

Standard evolutionary hypotheses accounting for the production of virulence factors assume that damage to the host is an unavoidable consequence of the extraction of resources from the host (Bull 1994; Frank 1996). Here, we offer an alternative scenario for the evolution of virulence factors. We hypothesize that a strategy of ‘proactive invasion’, simultaneously provoking and defending against a non-specific immune response, can allow a rare pathogen to invade and supplant a population of residents. The proactive invader wins by creating a novel environmental challenge to which it is already adapted.

We begin with a general ecological model predicting how and when virulent ‘proactive invaders’ (strain *V*) could invade new host environments that they are not adapted to by ‘changing the game’, i.e. changing their new environment to fit with their present adaptations and outcompeting resident commensals (strain *C*). We then build from the within-host scale to consider the ecological consequences for dynamics on the epidemiological (among-host) scale tracking hosts occupied by *C* or *V* (hosts *H*_*c*_ and *H*_*v*_). Turning to evolutionary considerations, we next illustrate that the two-trait nature of the ‘proactive’ strategy (provocation of, and adaptation to an environmental change) presents an evolutionary conundrum as neither trait alone is favoured in a homogeneous host population. Finally, we show that this conundrum is relaxed if host heterogeneity is introduced, with some hosts naturally presenting a more severe immunological environment, allowing the selection of more resistant strains (strain *D*) that then serve as a reservoir from which proactive invaders *V* can evolve. We relate our theoretical framework to recent empirical results on the immunological mediation of competition in diverse host–parasite systems.

## Model and results

### Within-host dynamics of proactive invaders

**Figure 1:**
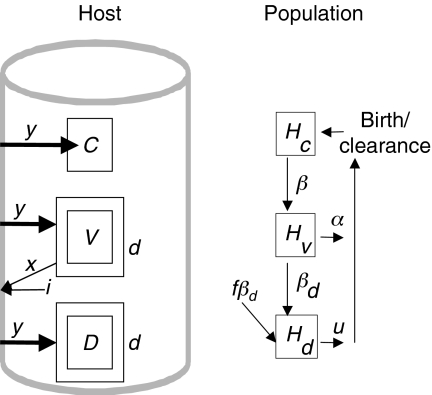
Schematic diagrams of the within-host and epidemiological models. (a) Within-host population-dynamical model. The three potential symbiont types, commensal, virulent and defended, are tracked by population densities *C*, *V* and *D*. Toxin production is represented by *x*, immuno-sensitivity by *y* and defence against an elevated immunological response by *d* (represented schematically by double-lines). The extent of intrinsic host inflammation is represented by *i*. (b) Epidemiological model. The host population consists of two intrinsically distinct types, *H* and *I* (‘healthy’ and ‘inflamed’). The focal *H* hosts can in principle be infected with any one of the strains, tracked by population densities *H*_c_, *H*_*v*_ and *H*_*d*_. The virulent strain *V* is characterized by transmission β and virulence or clearance α (in *H* hosts). The defended strain *D* in *H* hosts is characterized by transmission β_d_ and clearance *u*. Contact between *H* and *I* hosts is summarized by the parameter *f*.

Our within-host model focuses on the dynamics of a virulent ‘proactive invader’*V* competing with a resident commensal strain *C*, mediated by their shared host environment (Fig. 1a, given *D* = *i* =0). (1)
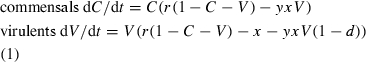
The commensal and virulent strains *C* and *V* have the same maximum rate of increase *r* and carrying capacity (normalized to 1). The host-provoking virulent bacteria make an additional investment *x* in provocation of the host’s inducible defences (e.g. inflammation), incurring a cost *x* and yielding an inflammatory response *yxV* (mediated by the immuno-sensitivity parameter *y*). Finally, the extent to which the virulent strain is better protected from the negative impact of the host’s inducible defence (relative to the commensal strain’s defences) is captured by the defence parameter *d.* Note that equation 1 can be translated into models of bacteriocin-mediated bacterial allelopathy (e.g. Frank 1994; Durrett & Levin 1997; Gordon & Riley 1999; Brown *et al.* 2006a) given the assumption of perfect resistance on the part of the ‘provoking’ strain, i.e. *d* =1. However, there is a significant difference of scale between bacteriocin-mediated and immuno-mediated allelopathy; in the case of immuno-mediation, a relatively small (and manipulative) investment *x* in killing behaviour can have a major environmental consequence *yxV* because of amplification by the relatively powerful host immune system (akin to amplification of temperate phage in virally mediated bacterial allelopathy, Brown *et al.* 2006a). As in the bacteriocin- and phage-mediated models cited above, a virulent (or ‘killer’) strain can potentially invade commensal (or ‘susceptible’) residents by modifying their shared environment (by triggering host inflammation, or releasing bacteriocins or phages), but how common must the virulent strain be to create an effective change?

A stability analysis of model 1 (following standard analyses of the Jacobian matrix; Otto & Day 2006) illustrates that the ability of commensals to stably exclude the virulent strain within a host depends on the initial inoculum of the virulent strain entering a new host. A population of pure commensals (*C* = 1, *V* = 0) is always locally stable (for positive *x*) against rare immigrants, whereas a population of pure virulents 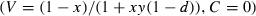
is also locally stable if *y*(*d*− *x*) > 1. If this latter condition holds, then a sufficiently large invading population of the virulent strain can create a sufficient level of host inflammation to outcompete its commensal rival in the newly degraded host environment. The critical inoculum size (relative to commensal resident population) is then given by (2)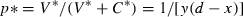 where 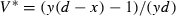
and 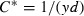
describe the repelling coexistence equilibrium. Invasion is favoured by low (but non-zero) provocation *x*, high defence *d* and high immuno-sensitivity *y*. Invasion and stable replacement of the commensal strain by the proactive strain *V* is only possible when the threshold p* is between 0 and 1, and *d* > *x* > 0. Given the ability of various inducible host defences to detect small changes and produce a big response (e.g. vertebrate immunity, behavioural fevers, etc.; Poitrineau *et al.* 2004), we can anticipate arbitrarily large values of *y* and thus p* can be arbitrarily small, facilitating invasion.

### Epidemiology of proactive invaders

Given the within-host dynamics described in the simple model above, what are the consequences for dynamics on the among-host (epidemiological) scale? To form a bridge with basic epidemiological theory, we use the within-host model to derive the basic epidemiological parameters governing virulence, clearance and transmission (Ganusov *et al.* 2002; Alizon & van Baalen 2005; Andre & Gandon 2006; Brown *et al.* 2006b; Gilchrist & Coombs 2006). Within host dynamics are governed by the inoculum threshold p*, which determines the success or failure of a potential transmission event (the pathogenic strain *V* is potentially transmitted to ‘susceptible’ hosts occupied by the commensal *C*). Specifically, we now assume that the transmission coefficient of the virulent strain β increases as the inoculum threshold p* decreases, and that host inflammation when occupied by the virulent strain drives the duration of infection 1/α (α equals the summed mortality and clearance rates) yielding (3)
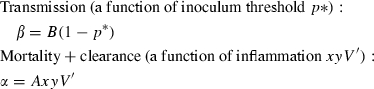
where *V* ′ is the equilibrium density of *V* bacteria once they have taken over the host 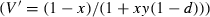, and *A* and *B* are weighting parameters. The transmission coefficient β captures the transmission benefits of proactive invasion, mediated by a reduced inoculum threshold. Conversely, the ‘mortality plus clearance’ rate α captures the costs of proactive invasion, in terms of reduced residency time within the host, mediated by increased inflammation. Increased inflammation could restrict residency of the virulent strain because of the increased clearance rates (recovery of the host) and/or increased mortality rates.

**Figure 2:**
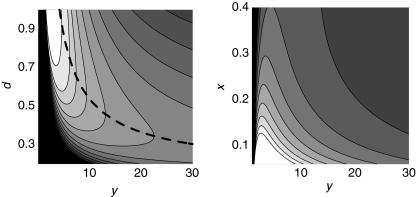
Epidemiology of the virulent and commensal strains. Reproductive number *R*_0_ of the virulent strain (in epidemiological competition with the commensal strain only) as a function of the within-host parameters toxin production *x*, additional immuno-defence *d* and immuno-sensitivity *y.* Contour lines represent increasing integer values of *R*_0_, from 1 to 15. In the black region *R*_0_< 1, and the virulent strain is excluded. (a) Parameters *m* = 5, *d* = 0.9. (b) Parameters *m* = 5, *x* = 0.1. Dotted line represents optimal immuno-defence d*.

Note that a key assumption of this approach is that the bistable within-host dynamics are much faster than the epidemiological dynamics, so that coexistence of strains within a single host can be neglected (i.e. the ‘superinfection’ limit of multiple infection assuming instantaneous replacement of strains, Nowak & May 1994; for a relevant empirical example see Kirkup & Riley 2004). The emergent α and β parameters can now be placed within the most basic epidemiological framework (equivalent to the model in Fig. 1b with *H*_*d*_ = *f* = 0) (4)
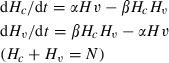
 Here, *H*_*c*_ and *H*_*v*_ represent the numbers of ‘susceptible’ (carrying the commensal strain *C* alone) and ‘infected’ (carrying the virulent strain *V* alone) hosts, which together sum to a static population size *N* (note we assume that any host mortality component of the α parameter is compensated by host births into the commensal class, ensuring a static host population). We can now derive from the microscopic (within host) behaviour, the key epidemiological value *R*_0_, the ‘reproductive number’ of an infection (Anderson & May 1991), which from (4) is *R*_0_ = *Nβ/α*. Substituting for β and α then yields (5)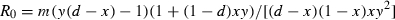 Here, *m* gathers the weightings *A*, *B* from eqn 3 and *N* from eqn 4. This *R*_0_ is plotted as a function of the within-host parameters *d*, *x* and *y* in Fig. 2.

Figure 2 illustrates that in the absence of a competent host immunity (i.e. as *y* tends to zero), the ‘proactive invader’ pathogen is unable to exploit its host. When *y* is too small, there is little inflammation to provoke, and the ‘virulent’ strain cannot spread. Therefore, we see that the success of the virulent strain is dependent on a competent immune system, highlighting the manipulative nature of this strategy. Note however that in contrast to the within-host scale, increasing *y* is not uniformly favourable to invasion. When *y* is overly large, the inflammation is so destructive that the infection is rapidly cleared (or the host dies). Thus, *R*_0_ is maximized for intermediate values of immuno-sensitivity *y*. Figure 2a also illustrates that for sufficiently elevated immuno-sensitivity *y*, an optimal level of bacterial defence *d* emerges (dashed line, Fig. 2a). Whereas on a within-host level, increasing *d* is uniformly favoured, on an epidemiological scale increasing *d* can be costly, as increased *d* leads to increased densities of the virulent strain *V* within a host and therefore ultimately to stronger inflammation and therefore more rapid clearance. Thus, the emerging trade-off governing *d* illustrates a potential case of a self-limiting infection, as big *d* would lead to large population and rapid clearance of the infecting lineage. Finally, Fig. 2b illustrates that *R*_0_ increases as immuno-provocation *x* tends to zero, reflecting the advantages of both decreased self-harm (if defence is imperfect) and decreased costs of provocation. However, note that *x* = 0 is not an optimum, as in the absence of any provocation, there is no inflammatory response to gain a competitive benefit from (see eqn 1). In the following sections we focus on the evolution of discrete (presence/absence) provocation and defence traits.

### Evolution of proactive invaders

We show above that a virulent proactive invader *V*, possessing both a trait modifying its local environment (for instance a toxin increasing host inflammation) and a trait conferring a superior adaptation to the modified environment (for instance, superior defence against oxygen-free radical or complement), can invade a commensal resident population (Fig. 2). However, the two-trait nature of the ‘proactive’ strategy of invasion presents an evolutionary conundrum, as neither carriage of the toxin-trait nor the defence-trait alone can supplant a local population of commensals, regardless of initial frequency. A toxin-alone strain *T* (equivalent to *V* with *d* = 0) is suicidal, increasing inflammation within a host (at a direct cost *x*), but without any relative advantage in face of the ensuing host response. Conversely, the superior defence of a defence-alone strain *D* (equivalent to *C* with *d >*0) is redundant in competition with non-provocative commensal strains, given any cost of a superior investment in defence.

This conundrum can be resolved by allowing some hosts (or some compartments within hosts) to present a tougher environment – for example, to be more inflamed – without exposure to toxins produced by a proactive virulent strain. Here, we consider that increased inflammation can also result from extrinsic factors (for instance due to differences in diet, shared parasites or to being a different host species), captured by the parameter *i* (the following is equivalent to the model in Fig. 1a, with *V* = 0) (6)
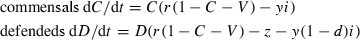
Now we have an increased level of inflammation *i* in the absence of any focal virulent strain. The defended strain *D* has an elevated level of defence *d* against this inflammation, which comes at a direct cost *z* (relative to costs of any defences already present in the commensal strain). Given *i* > *z/yd*, *D* will always out-compete *C* (conversely, when inflammation is closer to its baseline, i.e. *i* < *z*/*yd*, the additional defences are redundant, as outlined above). Thus, if some subset of a host population experience a sufficient extrinsic driver of inflammation (*i* > *z/yd*), then there will be selection for an increase in *d* among commensals exploiting this population.

Now, if an inflammation specialist lineage *D* was able to acquire a way to trigger host inflammation, e.g. a toxin, a proactively virulent strain *V* would be born, able to expand beyond the realms of extrinsically irritated hosts and into the wider population of hitherto un-inflamed hosts (see Fig. 2). Note that *V* would however be at a disadvantage in competition with *D* strains within already inflamed hosts, due to the cost of provoking inflammation, *x* and lack of any distinguishing adaptation to the environmental response (both *D* and *V* share the same elevated defence *d*). To simplify the dynamics of a system with distinct host types (potentially distinct species or individuals in distinct environments; naturally inflamed hosts with *i* > 0 and naturally uninflamed hosts with *i* = 0), we use a ‘source-sink’ style of approximation (Sokurenko *et al.* 2006). This approach allows us to assume that naturally inflamed hosts (which we call *I*) are a source for defended strain *D*, and a sink for all other strains (i.e. lineages of *C, T, V* tend to zero in this population of host, as they are either insufficiently defended or investing in a redundant provocation trait *x*).

If a *D* lineage was able to acquire (e.g. via horizontal gene transfer) a way to trigger host inflammation (i.e. recruit a toxin), a proactively virulent strain *V* would be born. We can then consider the fate of rare *V* mutants emerging from *D* strain parents (in *I*_*d*_ hosts) and arriving in *H* hosts (our focal *i* = 0 host population, see model 4, Fig. 1b). Figure 2 captured the basic scenario of epidemiological competition between *V* and *C* strains of bacteria in *H*_*c*_ and *H*_*v*_ hosts. We now consider the fate of more frequent *D* strain migrants moving from *I*_*d*_ to *H* hosts. Extending the previous epidemiological model 4 to allow for continued introduction of *D* bacteria from an *I*_*d*_ source, and transmission of both *D* and *V* bacteria among *H* hosts, we have the model described in Fig. 1b(7)
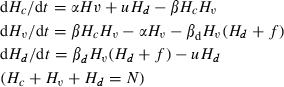
 where *H*_*d*_ is the number of *H* (*i* = 0) hosts carrying the defended *D* strain only. *H*_*d*_ hosts can infect inflamed *H*_*v*_ hosts with the *D* strain, with a transmission parameter β_*d*_ < β (we assume the rate of strain replacement is faster for the actively manipulative virulent strain). *H*_*v*_ hosts can also become infected with the *D* strain (becoming *H*_*d*_ hosts) by contact with *I*_*d*_ hosts. This second source of infection is parameterized by *f*, capturing both the density of *I* hosts and their contact rate with *H* hosts. When *H*_*d*_ = *f* = 0, the earlier epidemiological case in eqn 4 is recovered. Finally, *H*_*d*_ hosts are ultimately recolonized by the commensal strain (at rate *u*, where again *u*≤ β), once the inflammation triggered by the previous virulent occupant has subsided, thus *u* reflects the rate of decay of inflammation in the absence of further provocation.

**Figure 3:**
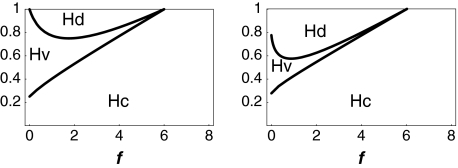
Equilibrium composition of ‘healthy’ (*H; i* = 0) host population, by infecting symbiont type, as a function of contact *f* with ‘inflamed’ host population (*I*_*d*_; *i* > 0). Figures illustrate stable coexistence equilibria of model 7 (Fig. 1b), for parameters α = 1, β = 4, β_*d*_ = 0.5. (a) Non-professional *D* (*u* = 1). Defended strain persists in the *H* population (*H*_*d*_ > 0) only due to continued introductions from *I*_*d*_. (b) Professionalized *D* (*u* = 0.25). The defended strain can persist by transmission among *H*_*v*_ hosts without continued introductions from *I*_*d*_.

Now the condition for *H*_*v*_ persistence (along with *H*_*c*_ and *H*_*d*_) becomes *Nβ* > α+ *f* β_*d*_. Figure 3 illustrates that the epidemiological persistence of proactive invaders (in *H*_*v*_ hosts) requires either low flow *f* of *D* from its *I* to *H* hosts, or a low rate of takeover β_*d*_ [relative to *R*_0_ (*H*_*v*_) = *Nβ/α*]. When combined flow and transmissibility *f* β_*d*_ is sufficiently high, the input of *D* can effectively swamp the *H*_*v*_ epidemic, excluding *V* from *H* hosts (except for rare arrivals of mutants from *D* source), maintaining the good health of the *H* population (ensuring *H*_*v*_ are excluded).

When the coexistence condition is fulfilled, the *V* strain (dominant in *H*_*v*_ hosts) coexists with the *D* strain (dominant in *H*_*d*_ hosts), however, it is not necessarily the case that *D* persists due to its ability to transmit from host-to-host among the *H* population. In Fig. 3a, the defended strain cannot persist in the *H* population without continued migrant flows from the *I*_*d*_ source population, as we assume that *H*_*d*_ hosts quickly loose their inflammation and then rapidly become *H*_*c*_ hosts. In contrast to Fig. 3b, we assume that ongoing inflammation following replacement by *H*_*d*_ is sufficiently long-lasting to allow the *D* strain to sustain itself in the *H* population purely by transmission among inflamed *H*_*v*_ and *H*_*d*_ hosts [if β_*d*_/*u* > *αβ/*(β− α)]. A consequence of this ‘professionalization’ (Antia *et al.* 2003; Sokurenko *et al.* 2006) is an even smaller market share for the *V* strain, as it is even more effectively parasitized by the non-provocative *D* strain.

**Figure 4:**
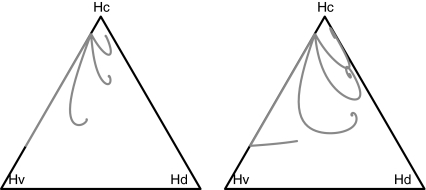
Dynamical approach to epidemiological equilibria of Fig. 3. Population dynamics of *H*_*c*_, *H*_*v*_ and *H*_*d*_ under model 7 with reference parameters in agreement with Fig. 3 (α = 1, β = 4, β_*d*_ = 0.5, *N* = 1). *H*_*c*_ ranges from 0 at the base of the triangle to 1 (dominance) at the top. Likewise *H*_*v*_ dominates in the bottom left corner and *H*_*d*_ dominates in the bottom right corner. (a) Parameters from Fig. 3a (*u* = 1, *H*_*d*_ maintained only by migration from *I*_*d*_ source). (b) Parameters from Fig. 3b (*u* = 0.25, potential for *H*_*d*_ to ‘professionalize’). All simulations initiated with relative densities of 0.9 *H*_*c*_, 0.09 *H*_*v*_ and 0.01 *H*_*d*_. Each grey line corresponds to a different value of *f* (0, 1, 3, 5). Higher values of *f* correspond to lower values of *H*_*c*_ at equilibrium (see Fig. 3).

The interactions between infection states *H*_*c*_, *H*_*v*_ and *H*_*d*_ display a non-transitive form of competitive advantage labelled ‘rock-paper-scissors’ after the popular children’s game (see e.g. Kerr *et al.* 2002); *H*_*v*_ beats *H*_*c*_, *H*_*d*_ beats *H*_*v*_, *H*_*c*_ beats *H*_*d*_, etc. In keeping with previous analyses of rock-paper-scissors models we find that for much of the parameter range supporting coexistence, the approach to the stable coexistence point is characterized by damped oscillations (Fig. 4). Akin to three-strain models of colicinogeny, we find population structure facilitates coexistence of sensitive, killer (toxin-antidote) and resistant (antidote-only) strains, however, it is noteworthy that in our model, coexistence of the three strains is not sustained by local dispersal (as in e.g. Kerr *et al.* 2002), but rather by a combination of metapopulation structure (Czaran & Hoekstra 2003) and source-sink dynamics.

## Discussion

### A general ecological model, relevant to parasites

We present a general ecological model of adaptive environmental change (Fig. 1). Related model approaches have been taken towards the evolution of flammability in plants (Kerr *et al.* 1999) and chemical (colicin) allelopathy in bacteria (Kerr *et al.* 2002; Czaran & Hoekstra 2003; Fitter 2003; Brown *et al.* 2006a). More generally, our ecological model relates to concepts of niche construction (Odling-Smee *et al.* 2003), ecosystem engineering (Jones *et al.* 1994) and the extended phenotype (Dawkins 1982). We argue that our model is particularly relevant to parasites, as they are in a position to manipulatively change their shared (immunological) environment cheaply and predictably, to be proactive invaders.

Turning to the evolution of proactive invaders, we present a potential evolutionary conudrum: why invest in increased defence (elevated *d*) in the absence of increased provocation, and why provoke (increased *x*) in the absence of elevated defence? Note that this conundrum only holds if defence *d* and provocation *x* are independent traits (then requiring host heterogeneity for its resolution). However, if a single underlying parasite trait caused a simultaneous increase in *d* and in *x*, the conundrum is avoided and a proactively virulent strain could evolve in a homogenous (*i* = 0) host population. Whether such a single underlying parasite trait exists remains to be seen, however, distinct genes separately coding for proinflammatory and anti-immune proteins (analogous to traits *x* and *d*) have been described in a diversity of pathogens (Hendrix *et al.* 2000; Bae *et al.* 2006; Shafikhani & Engel 2006). For instance, the notorious food-borne pathogen *Escherichia coli* strain O157:H7 carries both additional toxin and defence genes, relative to non-pathogenic *E. coli* strains (Plunkett *et al.* 1999; Hendrix *et al.* 2000; Ohnishi *et al.* 2001). Similarly, *Staphylococcus aureus*, a leading cause of hospital-acquired infections, secretes both varied toxins and factors neutralizing the innate immune response (Bae *et al.* 2006).

Turning to studies of within-host competition, a growing number of experiments point to a potentially widespread applicability of this model framework. For instance, it has been shown *in vivo* that *Haemophilus influenzae* can outcompete *Streptococcus pneumoniae* in the upper respiratory tract by recruiting and then activating neutrophils and complement (Lysenko *et al.* 2005). Similarly, more virulent strains of the rodent malaria parasite *Plasmodium chabaudi* experience a stronger within-host competitive advantage in immunocompetent hosts, relative to competition in immunodeficient hosts (Raberg *et al.* 2006). Most recently, it has been shown that host inflammatory responses triggered by *Salmonella enterica* serovar Typhimurium aid its ability to invade resident gut microbiota (Stecher *et al.* 2007). Testing for niche-emptying function in bacterial virulence genes may lead to medically significant reappraisals of host–parasite–parasite interactions across a diverse range of systems.

The present nested model contains a number of significant assumptions that merit further examination in future studies. There are several levels of within-host complexity that are currently overlooked, in particular the complexities of immunological dynamics (for examples of nested models with an explicit immunological dynamic, see e.g. Andre & Gandon 2006; Alizon and van Baalen 2005; Ganusov *et al.* 2002). A simple next step would be the addition to the model of an explicit shared environmental dimension (e.g. the degree of inflammation within a host compartment), which could potentially become dissociated from the dynamics of the strain that builds or triggers the environmental change (Brown *et al.* 2006a; Brown & Taddei 2007), thus calling into question the within-host assumption that the inflammatory response *yxV* is proportional to the density of the virulent strain *V* within the host. Another aspect of within-host complexity that demands further attention is the broader within-host parasite community, beyond strains experiencing direct resource competition within a specific host compartment. Our study focuses on competition among parasites sharing a similar niche, i.e. occupying similar locations and consuming similar resources within the host (Pedersen & Fenton 2007). Of course, any given host presents a diversity of distinct niches, and competitively driven immuno-manipulation in one host compartment is likely to have consequences mediated by the immune system beyond the competitive arena of the focal pathogens. These broader indirect effects are not captured in this study and as highlighted recently by several authors (Graham 2002; Poulin & Morand 2004; Graham *et al.* 2005; Pedersen & Fenton 2007) they are worthy of more attention. Finally, from an evolutionary perspective we have been focusing solely on immuno-manipulative strategies of invasion. Now we can ask, what are the potential (co)evolutionary responses of both the host and resident parasites or commensals in response to an immuno-manipulative proactive invader? Both the host and established commensals have a shared interest in mitigating the attempted niche ‘hijacking’ by virulent invaders (van Baalen & Sabelis 1995). Addressing these co-evolutionary ‘dangerous liaisons’ (van Baalen & Jansen 2001) among hosts, commensals and virulent parasites promises insights into the design of our immune systems and their exploitation by our varied symbionts.

### Evolution of virulence in a competitive environment

In recent years there has been increasing theoretical and experimental interest in the consequences of within-host competition on the evolution of parasite virulence, offering contrasting explanations for either an increase or decrease in virulence as competition increases (van Baalen & Sabelis 1995; Brown *et al.* 2002; West & Buckling 2003; Alizon 2006; Harrison *et al.* 2006). However, these frameworks all share the classic assumption that virulence factors are ultimately concerned with enhancing the exploitation of the host (Frank 1996), for instance liberating nutrients or providing shelter or dispersal. By viewing host-provoking toxins and immune resistance as tactics for gaining a competitive advantage within the digestive tract, we offer an alternative framework where toxin production may function to aid transfer of resources from commensal to pathogen, and not (just) from host to pathogen. Of course, these two functional hypotheses for toxin production are not exclusive, and it is even possible that a toxin that served initially as a tool to directly transfer resources from host to pathogen may subsequently serve, via an inflammatory immune response, as a tool of indirect immune-mediated interference competition. Importantly, even if the toxin precursor had a direct advantage to its producing parasite, its power as an agent of immunological provocation would not be raised by selection without an additional defensive trait already in place. Parasites are not necessarily constrained to manipulate the host immune system via damaging toxins; other potential tactics include the direct neutralization or skewing of the immune response (Riffkin *et al.* 1996; Graham 2002; Graham *et al.* 2005; Alizon 2006; Sansonetti & Di Santo 2007) including skewing the response against competitors by an established resident parasite (van Baalen & Sabelis 1995; Brown & Grenfell 2001). However, in an invasive setting, we argue that a rare pathogen is most likely to effectively generate a significant change in the immune environment by feeding the host a true ‘red alert’, a toxin.
